# Recent HIV-1 Infection Contributes to the Viral Diffusion over the French Territory with a Recent Increasing Frequency

**DOI:** 10.1371/journal.pone.0031695

**Published:** 2012-02-14

**Authors:** Pierre Frange, Laurence Meyer, Christiane Deveau, Laurent Tran, Cecile Goujard, Jade Ghosn, Pierre-Marie Girard, Philippe Morlat, Christine Rouzioux, Marie-Laure Chaix, for the French ANRS CO6 PRIMO Cohort Study Group

**Affiliations:** 1 Université Paris-Descartes, Equipe d'accueil 3620, Sorbonne Paris Cité, Paris, France; 2 Unité d'immunologie, hématologie et rhumatologie pédiatriques, Assistance Publique – Hôpitaux de Paris, Hôpital Necker – Enfants malades, Paris, France; 3 INSERM, Unité 1018, Université Paris-Sud, Faculté de Médecine Paris-Sud, Service d'Epidémiologie et de Santé Publique, Assistance Publique – Hôpitaux de Paris, Hôpital de Bicêtre, Le Kremlin-Bicêtre, France; 4 Service de Médecine interne, Assistance Publique – Hôpitaux de Paris, Hôpital de Bicêtre, Le Kremlin-Bicêtre, France; 5 Service de Maladies infectieuses et tropicales, Assistance Publique – Hôpitaux de Paris, Hôpital Saint Antoine, Paris, France; 6 Service de Médecine interne et maladies infectieuses, CHU de Bordeaux, Université Bordeaux Segalen, Bordeaux, France; 7 Laboratoire de virologie, Assistance Publique – Hôpitaux de Paris, Hôpital Necker – Enfants malades, Paris, France; Institut Pasteur, France

## Abstract

**Objective:**

To analyse the contribution of primary human immunodeficiency virus type 1 (HIV-1) infection (PHI) to the French viral epidemic.

**Methods:**

HIV-1 *pol* sequences included 987 PHI from the French ANRS PRIMO cohort between 1999 and 2010 and were analysed using a population-based phylogenetic approach. Clinical features, risk factors, sexual behaviour and drug resistance for clustered and nonclustered transmission events were ascertained.

**Results:**

Viruses from 125 (12.7%) of PHI cosegregated into 56 transmission chains, with increasing frequency during the last years (10.2% before 2006 versus 15.2% of clusters in 2006–2010, p = 0.02). The mean number of patients per cluster was 2.44. Compared to unique PHI, clusters involved more often men, infected through homosexual intercourse, of young age, with a high number of casual sexual partnerships and frequent previous HIV serological tests. Resistant strains were found in 16.0% and 11.1% of clusters and unique PHI, respectively (p = 0.11). Overall, 34% (n = 19) clusters included patients followed in French regions far apart, involving 13 clusters with at least one Parisian patient.

**Conclusions:**

PHIs are a significant source of onward transmission, especially in the MSM population. Recently infected people contribute to the spread of the viral epidemic throughout the French territory. Survey of transmitted drug resistance and behavioural characteristics of patients involved into clustered PHI may help to guide prevention and treatment interventions.

## Introduction

Understanding human immunodeficiency virus type 1 (HIV-1)-transmission dynamics is important in the design of effective prevention and treatment intervention. Some studies suggest that early stages of HIV infection may disproportionately contribute to viral transmission and spread of the epidemic [1]–[3]. Indeed, recent infections and particularly primary HIV infections (PHI) are associated with high viral burden in blood and semen, a major determinant of HIV transmission [1], [2], [4]–[6].

Different methods have been used to estimate the role of patients with acute infection in the spread of HIV-1. First, mathematical models using viral load/epidemiological/behavioural data have produced strikingly discrepant results, depending on the population studied and the assumptions used [7]–[18]. In these studies, the estimated percentage of new HIV cases caused by people with acute or early infection varied from less than 5% to more than 85%. Second, phylogenetic analysis of viral gene sequences has been used as a molecular epidemiological approach to reconstruct transmission events in early/acute infection [19]–[23]. Data from European and Canadian cohorts have reported significant clustering of viral sequences from 30–64% of recent infections [22], [24]–[27]. Interestingly, all these reports focused on patients living in quite confined but densely populated geographical areas, which could have had a significant impact on the frequency of clustering HIV transmissions.

The French National Agency for Research on AIDS (ANRS) PRIMO cohort established in 1996 is a national prospective study which allows to describe evolution of viral characteristics over calendar time, transmission risk factors and disease progression in patients enrolled at the time of PHI [28]–[32]. We planned to analyze the contribution of primary HIV infection to the French viral epidemic by a population-based phylogenetic approach of viral strains from 987 patients enrolled in the PRIMO cohort between 1999 and 2010.

## Methods

The Ethics Committee of Cochin Hospital approved the study, and all the patients gave their written informed consent. The next of kin, carers or guardians gave their written informed consent on the behalf of the enrolled minors/children.

Acute infection was defined as the period between exposure to the virus and completion of the initial immune responses, i.e. by detectable HIV RNA in plasma in the setting of a negative or indeterminate HIV antibody test. Primary and recent infections were defined as the period between 6 and 24 months following the exposure to the virus, respectively. Chronic infection was defined as evolving for more than 24 months after the viral exposure.

Transmission cluster was defined as a clade of patients infected with strains whose phylogenetic analysis revealed a strong homology, suggesting a reduced (or direct) transmission chain of the virus.

This study involved patients with primary HIV-1 infection (PHI) enrolled in the French ANRS PRIMO CO6 cohort between January 1999 and September 2010.

PHI was defined by a western blot profile compatible with ongoing seroconversion (incomplete western blot with absence of antibodies to pol proteins) in most patients (94%), detectable plasma HIV RNA with a negative or weakly reactive ELISA (2%), or an interval of less than 6 months between a negative and a positive ELISA result (4%), as previously described [33]. The date of infection was estimated as the date of symptom onset minus 15 days or, in asymptomatic patients, the date of incomplete western blot minus 1 month, or the midpoint between a negative and a positive ELISA result. Patients were enrolled if HIV infection was estimated to have occurred less than 6 months previously. At enrolment blood samples were collected for immunological and virological studies. All patients were antiretroviral (ART)-naïve at enrolment in the cohort. The present analysis included clinical epidemiological data and results of the serological screening for syphilis (*Treponema palladium* haemagglutination assay (TPHA) and Venereal Diseases Research Laboratory test (VDRL)), hepatitis B and hepatitis C viruses performed at enrolment. Participants completed standardized questionnaires describing HIV acquisition risk group, frequency of previous HIV screening tests and sexual behaviour (including number and characteristics of sexual intercourses before diagnosis of PHI and history of sexually transmitted infections (STI)).

HIV-RNA was quantified with the Cobas Amplicor HIV-1 Monitor 1.5 assay, the Cobas Taqman HIV-1 v1.0 and v1.5 assay (Roche Diagnostics, Meylan, France) or the Versant HIV-1-RNA 3.0 assay (Bayer Diagnostics, Emeryville, CA, USA), as recommended by the manufacturers. Both methods have a detection limit of 20 or 50 HIV-RNA copies/mL.

Drug resistance was evaluated by amplifying and sequencing the HIV-1 *reverse transcriptase* (RT) and *protease* genes in plasma HIV-RNA samples obtained at enrolment, as described elsewhere [29], [31]. Resistance to nucleoside RT inhibitors (NRTI), non-nucleoside RT inhibitors (NNRTI) and protease inhibitors (PI) was defined according to the 2010 ANRS HIV-1 genotypic resistance interpretation algorithm (www.hivfrenchresistance.org).

The RT nucleotide sequences were aligned with previously reported representatives of group M subtypes and circulating recombinant forms (CRFs) for which sequences are available in the HIV database (http://hiv-web.lanl.gov), using Clustal W (v1.7) with minor manual adjustments [34]. Phylogenetic trees were constructed with the neighbor-joining method [35], and reliability of the branching orders was implemented by Clustal W using the bootstrap approach [36]. Neighbor-joining plot was used to draw trees for illustrations.

Phylogenetic interrelationships among viral sequences were estimated using neighbor-joining trees [35] and maximum likelihood methods with BioEdit and MEGA4 integrated molecular evolutionary genetic analysis software [37], [38]. The existence of clusters was ascertained using the statistical robustness of the maximum likelihood topologies assessed by high bootstrap values (>98%) with 1000 resamplings and short branch lengths (genetic distances <0.015%) [19]. Infections in clusters were validated for congruent polymorphisms and mutational motifs.

Comparisons between clustered and unique PHI, and between patients involved into small versus large clusters were made by using the Chi-square or the Fisher's exact test for categorical variables, and the t-test or the Wilcoxon test for continuous variables. Logistic regression was performed to study the factors independently associated with belonging to a transmission cluster.

All RT nucleotide sequences were submitted to GenBank (accession numbers [JQ291804–JQ292793]).

## Results

A total of 987 patients were enrolled in the ANRS PRIMO cohort at the time of PHI in 81 different clinical sites between 1999 and 2010. Their baseline characteristics are summarized in Table 1. Patients were predominantly infected with viruses belonging to subtype B (n = 720, 72.9%) or CRF02_AG (n = 149, 15.9%). Overall, 15.5% of the patients were female. In comparison with men, women were more often patients of black ethnicity (32% versus 9%) and infected with non-B viruses (56% versus 22%). The only minor included in the study was a 17 year-old boy belonging with Caucasian ethnicity and infected through homosexual contact with a subtype B strain. One hundred and sixteen strains (11.7%) were resistant to at least one drug from one of the major three classes of antiretrovirals: 47 (4.8%) to NRTI, 41 (4.1%) to NNRTI, 58 (5.9%) to PI, 18 (1.8%) to two classes and 6 (0.6%) to three classes.

**Table 1:** Baseline characteristics of the 987 patients from the ANRS PRIMO cohort 1999–2010.

No (%) of male patients	834	(84.5%)
Median age (years) (range)	35	(17–79)
Ethnicity		
Caucasian	843	(85.4%)
African	126	(12.8%)
Asiatic	8	(0.8%)
Other	10	(1.0%)
Living place		
Paris area	515	(52.2%)
Other region of metropolitan France	448	(45.4%)
French overseas departments	24	(2.4%)
No (%) of patients in risk group		
Men who have sex with men	680	(68.9%)
Heterosexual	248	(25.1%)
Intravenous drug user	2	(0.2%)
Accidental exposure to infected blood	6	(0.6%)
Other	51	(5.2%)
Median CD4 cell count (cells/mm3) (range)	508	(22–1509)
Median HIV RNA (log10 copies/ml) (range)	5.11	(1.48–8.8)
Serological syphilis testing		
Isolated positive TPHA	116/877	(13.2%)
Isolated positive VDRL	63/885	(7.1%)
Positive TPHA and VDRL	57/871	(6.5%)
Serological HBV testing (n = 914)		
Positive HBs antigen	16	(1.8%)
Positive anti-HBc antibodies	238	(26.0%)
Isolated anti-HBs antibodies (vaccination)	360	(39.4%)
Positive serological HCV testing (n = 932)	21	(2.3%)

TPHA = *Treponema palladium* haemagglutination assay; VDRL = Venereal Diseases Research Laboratory test; HBV = hepatits B virus; HCV = hepatitis C virus.

**Figure 1:**
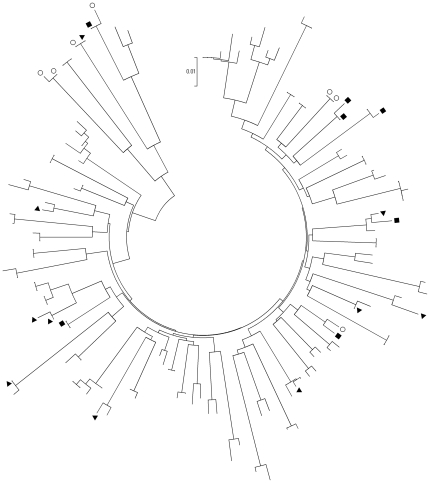
Evolutionary relationships of the 125 HIV-1 reverse transcriptase genes involved into cluster transmissions. The evolutionary history was inferred using the neighbour-joining method [35]. The optimal tree with the sum of branch length = 1.69802916 is shown. The tree is drawn to scale, with branch length in the same units as those of the evolutionary distances used to infer the phylogenetic tree. The evolutionary distances were computed using the Maximum Composite Likelihood method [37] and are in the units of the number of base substitutions per site. Codon positions included were 1^st^+2^nd^+3^rd^+noncoding. All positions containing gaps and missing data were eliminated from the dataset. There were a total of 408 positions in the final dataset. Phylogenetic analyses were conducted in MEGA4 [38]. Viruses isolated in women (○), male infected through heterosexual intercourse (▪) and patients (all were men) infected through unknown intercourse (▾) were represented.

Tree topology revealed that 125 (12.7%) PHIs segregated into 56 different transmission clusters, whereas the remaining infections represented unique sequences (Figure 1). Clustered transmission events included 2 to 5 infections per transmission cluster: 78.4% of clustered transmission chains had 2 infections per cluster. Overall, the mean number of patients per cluster was 2.44. Clusters were detected since the starting date of the cohort, but the frequency of clustered transmissions increased during the recent years (10.2% before 2006 versus 15.2% in 2006–2010, p = 0.02).

The maximum window periods for transmission after PHIs were estimated by determining the time interval between the first and last infection within each cluster. Transmission intervals ranged from 0 to 85.5 months with a median transmission interval of 15 months (IQR 5–27.5). When considering patients with the closest dates of diagnoses as (overlapping) pairs in clusters with more than two patients, the median pairwise tranmission intervals was 10.5 months. The maximum period of time between the first and the latest PHI within the same cluster was ≤6 months in 17/56 (30.4%) and ≤24 months in 39/56 (69.6%) clustered events. When restricting the analysis to the recent years (2006–2010), this maximum period of time was ≤6 months in 21.9% and ≤24 months in 64.1% of clustered transmissions. Large clusters, including ≥4 patients, tended to have a longer time length than clusters of smaller size (19.3 versus 12.0 months, p = 0.33). Finally, large clusters tended to be more frequent in recent years compared with small clusters (5 patients involved in a large cluster before 2006 versus 13 patients in 3 large clusters in 2006–2010), but the difference was not significant (p = 0.20).

The clinical, immunological, virological and behavioural characteristics were compared between clustered and unique PHI (Table 2). Clusters involved more frequently men (94.4% vs 83%, p = 0.001), of younger age (33 vs 35 years, p = 0.01), belonging to Caucasian ethnicity (89.6% vs 84.8%, p = 0.16), infected through homosexual intercourse (87.3% vs 80.6%, p = 0.08) and with a higher number of casual sexual partnerships within the last 6 months (p = 0.004) than unique PHI. The number of previous HIV serological tests tended to be higher in patients involved into clustered transmissions compared to the others (p = 0.14). The proportion of men who have sex with men (MSM), heterosexual men and women involved into clusters was 15.1%, 6.8% and 4.5%, respectively.

**Table 2:** Comparison of the characteristics of patients included into clustered transmission versus non-clustered: the ANRS PRIMO cohort 1999–2010.

Characteristics	Clustered transmissions (n = 125)		Nonclustered unique PHI (n = 862)		p
Male	118	94.4%	716	83%	0.001
Median age (years) (range)	33	19–64	35	17–79	0.01
Caucasian ethnicity	112	89.6%	731	84.8%	0.16
Living place in Paris area	61	48.8%	454	52.7%	0.42
Risk group: MSM	103	87.3%	577	80.6%	0.08
Viral subtype B	106	84.8%	614	71.2%	0.001
Viral resistance					
≥1 class of antiretrovirals	20	16%	96	11.1%	0.11
≥2 classes of antiretrovirals	0	0	24	2.8%	0.06
Median CD4 cell count (cells/mm3) (range)	504	62–1265	509	22–1509	0.61
Median HIV RNA (log10 copies/ml) (range)	5.13	2.2–7.41	5.11	1.48–8.08	0.36
Year of inclusion					
2006–2010	73	58.4%	406	47.1%	0.02
Serological syphilis testing at baseline					
Isolated positive TPHA	14	12.4%	102	13.4%	0.78
Both positive TPHA and VDRL	7	6.2%	50	6.6%	0.89
Prior sexually transmitted infections (1)	31	26.3%	196	24.1%	0.6
Median number of previous negative HIV serological tests (range)	5	0–30	3	0–72	0.14
Frequency of previous HIV screening					
>1 test/year	53	61.6%	279	52.7%	0.13
Number of regular sexual partnerships(2)					
0–1	80	77.7%	506	78.2%	
2	8	7.8%	68	10.5%	0.48
>2	15	14.6%	73	11.3%	
Number of casual sexual partnerships(2)					
0–1	28	28.3%	291	45.4%	
2–10	45	45.5%	205	31.9%	0.004
>10	26	26.3%	147	22.9%	

Note: the total of patients for each variable does not always equal the total sample due to some missing values. PHI = primary HIV infection; MSM = men having sex with men; TPHA = *Treponema palladium* haemagglutination assay; VDRL = Venereal Diseases Research Laboratory test.

(1) Urethritis, rectitis, genital herpes infection, vulvo-vaginal candidosis, condyloma and/or syphilis.

(2) In the last 6 months preceding PHI diagnosis.

Forty nine per cent of the patients involved into transmission clusters were followed in Paris area (61/125, 48.8%). However, the proportion of Parisian patients involved into clusters was not different from the proportion of patients followed outside the Paris area (11.8% vs 1.6%, p = 0.41). Overall, two-thirds (n = 37) of the clusters exclusively comprised patients followed in the same geographical area: 20 clusters (43 patients) in the Paris area and 17 clusters (38 patients) in other French regions. The other third of the clusters involved 44 patients living in French regions far apart: 13 out of these 19 clusters comprised at least one Parisian patient.

The strains isolated in clustered events belonged to subtype B (n = 106), CRF02_AG (n = 4), subtype D (n = 2) or CRF01_AE (n = 2); the remaining 11 viruses were unique recombinant forms, but 5 of them strongly clustered with a previously characterized B/C/U recombinant virus [39]. Seven women were included into transmission clusters, of which 4 were infected with non-B subtype strains. Overall, infection with a B subtype strain was more frequent in clusters than in unique PHI (84.8% vs 71.2%, p = 0.001), mainly because clusters involved more often French MSM; this difference was not found when restricting the analysis to the MSM population: 87.4% versus 82.7% of B subtype viruses in clustered and non-clustered events, respectively (p = 0.24).

Overall, 20/125 (16%) strains involved into clustered events were resistant to at least one drug from the three major classes of ART : 2 (1.6%) to NRTI, 4 (3.2%) to NNRTI, 14 (11.2%) to PI. Interestingly, 18/20 of these strains (90%) were isolated in patients whose PHI occurred between 2006 and 2010. When performing the same analysis in unique PHIs, only 48/96 (50%) of the resistant strains were isolated in 2006–2010. No strain isolated in clusters was resistant to >1 class of ART, compared with 2.8% in unique PHI (p = 0.06). Finally, the median baseline CD4 cell count and HIV RNA were similar in clustered and non clustered transmissions.

We also compared the characteristics of the patients involved into small (2–3 PHI/cluster) or large clusters (4–5 PHI/cluster) (Table 3). In keeping with what we found when comparing clustered transmissions and unique PHI, patients in large clusters tended to be more often MSM and infected since 2006, to be younger, to have a higher number of regular and/or casual sexual partnerships within the last 6 months, to report more previous HIV negative tests, but the differences were not all significant. Similar trends were found when restricting this analysis to the MSM population. Large clusters exclusively comprised MSM. None of them had both positive TPHA and VDRL syphilis serological tests (versus 7.3% in small clusters, p = 0.59), neither was infected with a resistant strain (versus 18.7% in small clusters, p = 0.07).

**Table 3:** Comparison between the characteristics of the patients included into small (2–3 patients/cluster) or large (>4 patients/clusters) clustered events.

Characteristics	Large clusters (≥4 PHI) (n = 18)		Small clusters(2–3 PHI) (n = 107)		p
Size of the clusters (months) (range)	19.3	10–53	12.0	0–85.5	0.33
Male	18	100%	100	93.5%	0.59
Median age (years) (range)	32	24–44	35	19–64	0.13
Caucasian ethnicity	17	94.4%	95	88.8%	0.69
Living place in Paris area	11	61.1%	50	46.7%	0.26
Risk group: MSM	18	100%	85	85%	0.12
Viral subtype B	13	72.2%	93	86.9%	0.15
Viral resistance ≥1 class of antiretrovirals	0	0	20	18.7%	0.07
Median CD4 cell count (cells/mm3) (range)	570	128–843	479	62–1265	0.92
Median HIV RNA (log10 copies/ml) (range)	4.99	2.93–6.67	5.14	2.2–7.41	0.58
Year of inclusion					
2006–2010	13	72.2%	60	56.1%	0.20
Serological syphilis testing at baseline					
Isolated positive TPHA	1	5.9%	13	13.5%	0.69
Both positive TPHA and VDRL	0	0	7	7.3%	0.59
Past sexually transmitted infections(1)	5	29.4%	26	25.7%	0.77
Median number of previous negative HIV serological tests (range)	7	1–30	3.5	0–25	0.01
Frequency of previous HIV screening					
≤1 test/year	9	56.3%	48	68.6%	0.35
Number of regular sexual partnerships(2)					
0–2	10	62.5%	70	80.5%	0.19
>2	6	37.5%	17	19.5%	
Number of casual sexual partnerships(2)					
0–1	3	18.8%	25	30.1%	0.55
≥2	13	81.3%	58	69.9%	

Note: the total of patients for each variable does not always equal the total sample due to some missing values.

(1) Urethritis, rectitis, genital herpes infection, vulvovaginal candidosis, condyloma and/or syphilis.

(2) In the last 6 months preceding PHI diagnosis.

## Discussion

This study involved phylogenetic analysis of 987 HIV-1 strains isolated from patients diagnosed at the time of PHI between 1999 and 2010. To the best of our knowledge, this is one of the largest studies performed to date in HIV-1 primary infection. The proportion of 12.7% of patients involved into clustered events is lower than the rate of 30–64% of clusters in previous reports from others geographical regions [22], [24]–[27]. However, both the populations and the methods of these phylogenetic studies are heterogeneous: definition of recent infection varied from 6 to 18 months after infection, analysis was restricted to PHI or included also chronically infected patients, sample sizes renged from 130 to 1150 patients. In addition, timing and duration of inclusion (from 5 years over 1999–2003 to 12 years over 1998–2009) and routes of HIV infection (0–28% of intravenous drugs users, 57–100% of MSM) were different. It is difficult, thus, to extrapolate the results from one study to another. Besides, these reports focused on patients living in densely populated but quite restricted areas, which could have facilitated the identification of clustered events in the studied population. The setting is different in the PRIMO cohort: participants are scattered throughout the French territory and half of them are living outside the Paris area. Such characteristics allowed us to underscore the role of recent HIV infections in the spread of the viral epidemic at the national level; this could also explain the lower frequency of clusters in our report than in previous phylogenetic studies. A well known and acknowledged limitation comes from the fact that many individuals remain undiagnosed and unaware of their HIV status in France [40]. Because patients not enrolled in the PRIMO cohort may have been involved into clustered events, 12.7% should be considered as a minimal estimation of the prevalence of clusters among PHI in France.

Particular characteristics of patients involved into clusters events have been evidenced in our study: gender, as shown in the Swiss cohort [26], a homosexual preference, as previously described [22], a younger age, as suggested in the Pao's study [22] but not in the Canadian and Swiss reports [24], [26] and a high number of casual sexual partnerships. These results underline behavioural differences in HIV-risk sexual practices between men/women and younger/older individuals. Interestingly, neither a history of STI nor a positive syphilis serological test at enrolment was more frequent in clustered events. However the frequency of these STI was notably high in both groups of patients, suggesting a high frequency of unprotected sex in the whole population: around one in four patients declared having suffered from at least one STI and near one in seven individuals had a positive syphilis serological test. Moreover, half of these positive tests were probably due to an active infection at the time of PHI (both positive TPHA and VDRL tests).

In the PRIMO cohort, we found a significant increase in clustered transmission in the recent years, involving 15.2% of the enrolled patients in 2006–2010 versus 10.2% before. This result could be explained by the failure to control the HIV epidemic in the MSM population. Indeed, recent studies have highlighted the high HIV incidence and the increasing number of new HIV diagnoses in MSM in France as well as in several other industrialised countries [41]–[43]. Several factors could explain this evolution: an increase in unprotected anal sex and number of sexual partners in MSM with and without HIV infection [44], an increase in transmission of primary and secondary syphilis and rectal lymphogranuloma venereum [45] and a high HIV prevalence in the MSM population [41]. The increasing frequency of HIV-risk sexual practices in MSM may have led to raise the frequency of clustered events.

We found a relatively low (11.7%) proportion of transmitted-drug resistant (TDR) variants. Furthermore, although transmission of multi-drug resistant (MDR) strains has been evidenced in the PRIMO cohort [46], no MDR viruses were involved in clustered transmissions. Different results were reported on the frequency of TDR in clustered infections. Yerly et al. have recently described in newly diagnosed HIV infections that clusters were more frequent in individuals with resistant variants than in those with sensitive strains; they concluded that newly diagnosed, untreated individuals were a significant source of onward transmission, particularly of resistant viruses, thus suggesting an important self-fueling mechanism for TDR [26]. In contrast, other reports have described a lower prevalence of TDR in clusters as compared with unique PHI, suggesting a reduced transmissibility of drug-resistant strains, and in particular of MDR variants [23], [24]. To sum up, there is no evidence of greater or lower transmissibility of resistant variants. However, in population with high rates of TDR and/or frequent HIV-risk behaviour leading to high proportion of clustered events, clusters could contribute to the spread of resistant strains in the population.

We found that the maximal period of time between the first and the latest PHI within the same cluster was ≤6months and ≤24 months in 27.2% and 68.8% of the clusters,, respectively. These results are in keeping with previous phylogenetic studies, showing that the majority of transmission events occurred within the 2 years following PHI [24]–[27], but only part of them could be rigorously interpreted as acute-to-acute infections [47]–[49]. Although all patients of the cohort have been enrolled at the time of PHI, most of them contracted the virus from chronically infected patients and the role of “recent chronic” (but not necessarily primary) infections has increased in the recent years. This finding is important to design effective preventive strategies in order to reduce HIV transmission, especially to assess the potential benefit for public health of an early antiretroviral therapy. A recent Swiss report showed lower viral transmission rates in HIV-infected MSM who received an ART since the time of PHI [50]. However, the long period of time (>2 years) between the first and latest patients within the same cluster in more than 30% of the cases, may suggest the need for a, not only early, but also prolonged ART to reduce the risks of HIV spread in the population. Interestingly, the proportion of early-treated patients fell from 88% in 1996–1997 to 29% in 2006–2007 in our cohort (Troude P, AIDS 2009) and we can not exclude a possible causal link between this evolution and the recent increasing frequency of clustered transmissions. Of note, French guidelines do not recommend the discontinuation of a combined ART initiated at the time of PHI [51].

In our study, more than 75% of the clustered transmissions chains had 2 infections per cluster. Unlike the results by Brenner et al [24], [27], the proportion of large clusters (≥4 PHI) was significantly lower in our study (1.8% of all PHI). As previously discussed, the geographical proximity of the participants enrolled in the Quebec studies, including mainly MSM living in Montreal area, may have increased the risk that many people are infected by the same strain. We also analysed differences among patients involved into small and large clusters. Although the small number of large clusters limited the possibilities of finding statistically significant differences, we did evidenced some specific features. Large clusters involved exclusively MSM; they tended to be younger and to have a higher number of declared sexual partners, and a higher number of previous HIV tests. Interestingly, the proportion of positive syphilis serological tests was lower in large versus small clusters. These findings may be explained by one behavioural assumption, which has been recently evoked in the Montreal MSM epidemic: Brenner et al. concluded that MSM in small clusters engaged in higher HIV risk contacts but with fewer partners than those involved into large clusters [27]. Conversely, patients involved into large clusters may be engaged with a great number of sexual partnerships, perhaps in meeting places for sex with multiple partners, but highly informed about screening, condoms and information about HIV transmission. This could explain the low prevalence of positive syphilis serology and the high frequency of HIV screening in this group. If this assumption is confirmed in further behavioural studies, it could have a major impact in understanding the different dynamic transmission profiles to be addressed for specifically targeted prevention and/or antiretroviral treatment interventions.

To conclude, we show evidence that 12.7% of patients diagnosed at the time of PHI were involved into clustered transmissions in France. Given than many individuals remain undiagnosed and unaware of their HIV status in France, this 12.7% rate should be considered as a minimal estimation of the frequency of clustered transmissions in France. Such transmission events, whose frequency increased in the most recent years, have a significant impact on the spread of the HIV epidemic, with a specially high transmission risk during the 2 years following infection. Our findings may suggest that starting antiretroviral therapy at the time of PHI may limit HIV transmission, especially in young MSM with high number of sexual partnerships, who are usually involved into large clustered events. Moreover, clusters have the potential to contribute to the spread of resistant strains. Our study underscores recommendations for genotyping in primary infection to document clustering of infection and provide information on transmitted drug resistance and behavioural characteristics of infected people.
